# 1,3-Bis[(naphthalen-2-ylsulfan­yl)meth­yl]benzene

**DOI:** 10.1107/S1600536812015280

**Published:** 2012-04-18

**Authors:** Esteban Padilla-Mata, Juan M. German-Acacio, Marco A. García-Eleno, Reyna Reyes-Martínez, David Morales-Morales

**Affiliations:** a Instituto de Química, Universidad Nacional Autónoma de México, Circuito exterior, Ciudad Universitaria, México, DF 04510, Mexico; b Ciencias Básicas e Ingeniería, Recursos de la Tierra, Universidad Autónoma Metropolitana, Av. Hidalgo Poniente, La Estación Lerma, Lerma de Villada, Estado de México, CP 52006, Mexico

## Abstract

Mol­ecules of the title compound, C_28_H_22_S_2_, are located on a crystallographic mirror plane with one half-mol­ecule in the asymmetric unit. The dihedral angle between the phenyl ring and the naphthyl unit is 83.14 (7)°. In the crystal, mol­ecules are inter­connected by C—H⋯S and C—H⋯π inter­actions.

## Related literature

For information on pincer compounds, see: Albrecht & Morales-Morales (2009 ▶); Arroyo *et al.* (2003 ▶); Morales-Morales (2004 ▶, 2008 ▶, 2009 ▶); Morales-Morales & Jensen (2007 ▶).
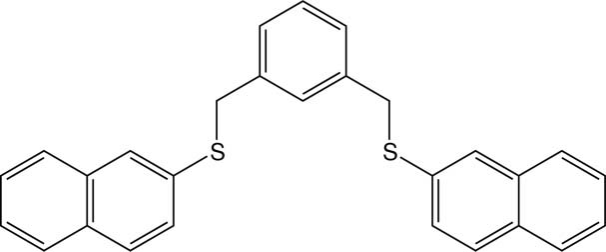


## Experimental

### 

#### Crystal data

C_28_H_22_S_2_
*M* *_r_* = 422.58
Orthorhombic, 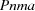

*a* = 8.651 (2) Å
*b* = 41.235 (10) Å
*c* = 6.0517 (14) Å
*V* = 2158.9 (9) Å^3^
*Z* = 4
Mo *K*α radiation
μ = 0.26 mm^−1^
*T* = 298 K
0.48 × 0.42 × 0.07 mm

#### Data collection

Bruker SMART APEX CCD diffractometer
Absorption correction: analytical (*SADABS*; Bruker; 2007 ▶) *T*_min_ = 0.893, *T*_max_ = 0.979
7907 measured reflections
1994 independent reflections
1345 reflections with *I* > 2σ(*I*)
*R*_int_ = 0.065

#### Refinement

*R*[*F* ^2^ > 2σ(*F* ^2^)] = 0.054
*wR*(*F* ^2^) = 0.124
*S* = 1.02
1994 reflections
139 parameters
H-atom parameters not refined
Δρ_max_ = 0.21 e Å^−3^
Δρ_min_ = −0.16 e Å^−3^

### 

Data collection: *SMART* (Bruker, 2007 ▶); cell refinement: *SAINT* (Bruker, 2007 ▶); data reduction: *SAINT*; program(s) used to solve structure: *SHELXTL* (Sheldrick, 2008 ▶); program(s) used to refine structure: *SHELXTL*; molecular graphics: *SHELXTL*; software used to prepare material for publication: *SHELXTL*.

## Supplementary Material

Crystal structure: contains datablock(s) I, global. DOI: 10.1107/S1600536812015280/bt5855sup1.cif

Structure factors: contains datablock(s) I. DOI: 10.1107/S1600536812015280/bt5855Isup2.hkl

Supplementary material file. DOI: 10.1107/S1600536812015280/bt5855Isup3.cml

Additional supplementary materials:  crystallographic information; 3D view; checkCIF report

## Figures and Tables

**Table 1:** Hydrogen-bond geometry (Å, °) *Cg*1 and *Cg*2 are the centroids of the C1–C4/C2′/C3′ and C6–C9/C14/C15 rings, respectively.

*D*—H⋯*A*	*D*—H	H⋯*A*	*D*⋯*A*	*D*—H⋯*A*
C5—H5*B*⋯S1^i^	0.97	2.86	3.806 (4)	164
C1—H1⋯*Cg*1^i^	0.93	2.94	3.867 (4)	173
C13—H13⋯*Cg*2^ii^	0.93	2.76	3.503 (3)	138

Symmetry codes: (i) 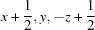; (ii) 
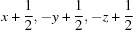
.

**Fig. 1.**
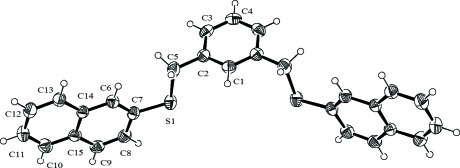
The molecular structure of the title compound with displacement ellipsoids at the 40% probability.

**Fig. 2.**
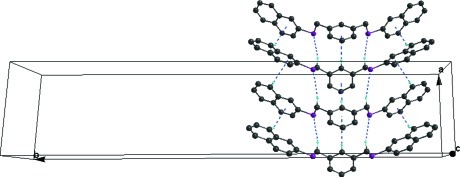
The title compound is linked by C—H···S and C—H···π intermolecular interactions along the a axes, the hydrogen atoms for the interactions are drawn.

## supplementary crystallographic information

### Comment

Pincer compounds represent a group of species with very particular and interesting properties among which their high thermal stability and unusual reactivities that confer to the metal complexes they form stand out. It is due, to the characteristics of robustness and thermal stability that pincer compounds have attracted the continuos attention of the chemistry community for multiple applications (Morales-Morales *et al.*, 2004, Morales-Morales *et al.* 2007, Albrecht *et al.*, 2009, Morales-Morales, 2008, Morales-Morales, 2009). In the beginning, the very simple backbone exhibited by these compounds did not anticipate the wide variety of possible functionalization in the main frame of the complex.

Among these species, those including sulfur as donor atom have been scarcely studied (Arroyo *et al.*, 2003), mostly due to the well known tendency of sulfur to kill the activity of homogeneous catalysts. Thus, following our continuous interest in the synthesis of pincer type ligands we report the crystal structure of the potentially pincer sulfur based ligand 1,3-bis((naphthalen-2-ylthio)methyl)benzene.

In the asymmetric unit only half of the molecule of the compound 1,3-bis(naphthalen-2-ylthio)methyl)benzene is found. The other half is generated by a mirror plane. The molecular structure of the title compound is shown in Figure 1. The phenyl and the naphthyl enclose a dihedral angle of 83.14 (7)°. The two naphthyl planes have a dihedral angle of 45.64 (4)°. The sulfur atoms form weak hydrogen bonds (C5—H5···S1). Two C—H···π interactions [C1—H1···*Cg*1 and C13—H13···*Cg*2] further connect the molecules into ribbons running along the *a*-axis

### Experimental

To a solution of 2-naphthalenethiol (0.320 g, 2.0 mmol), 0.057 g (2.5 mmol) of NaH in toluene (100 ml) were added. The reaction mixture was stirred at room temperature for 3 h. After this time, 0.264 g (1 mmol) of 1,3-bis(bromomethyl)benzene were added to yield a colourless solution that was further stirred for 5 h. Then, the solvent was evaporated under vacumm affording 1,3-bis[(naphthalen-2-ylsulfanyl)methyl]benzene (0.24 g) as a microcrystalline white powder (93% based on 1,3-Bis(bromomethyl)benzene). mp: 120–122 °C, MS—EI (m/*z*): 422 (100%) [*M*]^+^, ^1^H-NMR (300 MHz, CDCl_3_) δ (p.p.m.): 4.05 (s, 4H), 7.06–7.12 (d, 3H), 7.22 (s, 1H), 7.24 (d, 1H), 7.27 (d, 1H), 7.29–7.39 (m, 4H), 7.54–7.70 (m, 2H). 13 C-NMR (757 MHz, CDCl3) δ (p.p.m.): 38.82, 125.79, 126.50, 127.22, 127.72, 127.81, 127.85, 128.36, 128.75, 129.45, 133.72, 133.76, 137.73.

### Refinement

H atoms were included in calculated positions (C—H = 0.93 Å for aromatic H, C—H = 0.97 Å for methylene H), and refined using a riding model, with *U*_iso_(H) = 1.2*U*_eq_ of the carrier atom.

### Crystal data

**Table:**

C_28_H_22_S_2_	*F*(000) = 888
*M**_r_* = 422.58	*D*_x_ = 1.300 Mg m^−^^3^
Orthorhombic, *P**n**m**a*	Mo *K*α radiation, λ = 0.71073 Å
Hall symbol: -P 2ac 2n	Cell parameters from 2418 reflections
*a* = 8.651 (2) Å	θ = 3.0–25.1°
*b* = 41.235 (10) Å	µ = 0.26 mm^−^^1^
*c* = 6.0517 (14) Å	*T* = 298 K
*V* = 2158.9 (9) Å^3^	Plates, colorless
*Z* = 4	0.48 × 0.42 × 0.07 mm

### Data collection

**Table:**

Bruker SMART APEX CCD diffractometer	1994 independent reflections
Radiation source: fine-focus sealed tube	1345 reflections with *I* > 2σ(*I*)
Graphite monochromator	*R*_int_ = 0.065
Detector resolution: 0.661 pixels mm^-1^	θ_max_ = 25.4°, θ_min_ = 3.0°
ω–scans	*h* = −10→9
Absorption correction: analytical (*SADABS*; Bruker; 2007)	*k* = −43→48
*T*_min_ = 0.893, *T*_max_ = 0.979	*l* = −7→7
7907 measured reflections	

### Refinement

**Table:**

Refinement on *F*^2^	Primary atom site location: structure-invariant direct methods
Least-squares matrix: full	Secondary atom site location: difference Fourier map
*R*[*F*^2^ > 2σ(*F*^2^)] = 0.054	Hydrogen site location: inferred from neighbouring sites
*wR*(*F*^2^) = 0.124	H-atom parameters not refined
*S* = 1.02	*w* = 1/[σ^2^(*F*_o_^2^) + (0.0535*P*)^2^] where *P* = (*F*_o_^2^ + 2*F*_c_^2^)/3
1994 reflections	(Δ/σ)_max_ < 0.001
139 parameters	Δρ_max_ = 0.21 e Å^−^^3^
0 restraints	Δρ_min_ = −0.16 e Å^−^^3^

### Special details

**Table:**

Geometry. All e.s.d.'s (except the e.s.d. in the dihedral angle between two l.s. planes) are estimated using the full covariance matrix. The cell e.s.d.'s are taken into account individually in the estimation of e.s.d.'s in distances, angles and torsion angles; correlations between e.s.d.'s in cell parameters are only used when they are defined by crystal symmetry. An approximate (isotropic) treatment of cell e.s.d.'s is used for estimating e.s.d.'s involving l.s. planes.
Refinement. Refinement of *F*^2^ against ALL reflections. The weighted *R*-factor *wR* and goodness of fit *S* are based on *F*^2^, conventional *R*-factors *R* are based on *F*, with *F* set to zero for negative *F*^2^. The threshold expression of *F*^2^ > σ(*F*^2^) is used only for calculating *R*-factors(gt) *etc*. and is not relevant to the choice of reflections for refinement. *R*-factors based on *F*^2^ are statistically about twice as large as those based on *F*, and *R*- factors based on ALL data will be even larger.

### Fractional atomic coordinates and isotropic or equivalent isotropic displacement parameters (Å^2^)

**Table:**

	*x*	*y*	*z*	*U*_iso_*/*U*_eq_	
S1	0.49764 (9)	0.180146 (17)	0.07221 (12)	0.0516 (3)	
C1	0.5547 (5)	0.2500	0.3529 (6)	0.0456 (10)	
H1	0.6393	0.2500	0.2581	0.055*	
C2	0.4932 (3)	0.22086 (6)	0.4217 (4)	0.0432 (7)	
C3	0.3680 (4)	0.22111 (7)	0.5638 (4)	0.0489 (8)	
H3	0.3254	0.2017	0.6123	0.059*	
C4	0.3065 (5)	0.2500	0.6332 (6)	0.0533 (11)	
H4	0.2221	0.2500	0.7285	0.064*	
C5	0.5635 (4)	0.18939 (6)	0.3482 (5)	0.0561 (8)	
H5A	0.5335	0.1722	0.4484	0.067*	
H5B	0.6754	0.1911	0.3498	0.067*	
C6	0.6690 (3)	0.12415 (6)	0.1558 (4)	0.0419 (7)	
H6	0.7048	0.1337	0.2853	0.050*	
C7	0.5702 (3)	0.14102 (6)	0.0228 (4)	0.0417 (7)	
C8	0.5192 (3)	0.12636 (7)	−0.1764 (4)	0.0476 (7)	
H8	0.4513	0.1376	−0.2677	0.057*	
C9	0.5670 (4)	0.09654 (7)	−0.2359 (4)	0.0491 (8)	
H9	0.5331	0.0877	−0.3688	0.059*	
C10	0.7184 (4)	0.04703 (7)	−0.1541 (5)	0.0560 (8)	
H10	0.6865	0.0377	−0.2863	0.067*	
C11	0.8133 (4)	0.03010 (7)	−0.0168 (5)	0.0603 (9)	
H11	0.8453	0.0093	−0.0553	0.072*	
C12	0.8624 (4)	0.04379 (7)	0.1811 (5)	0.0558 (8)	
H12	0.9270	0.0321	0.2744	0.067*	
C13	0.8166 (3)	0.07420 (6)	0.2392 (5)	0.0474 (7)	
H13	0.8505	0.0830	0.3720	0.057*	
C14	0.7181 (3)	0.09256 (6)	0.1010 (4)	0.0392 (6)	
C15	0.6678 (3)	0.07843 (6)	−0.0998 (4)	0.0426 (7)	

### Atomic displacement parameters (Å^2^)

**Table:**

	*U*^11^	*U*^22^	*U*^33^	*U*^12^	*U*^13^	*U*^23^
S1	0.0523 (5)	0.0423 (4)	0.0602 (5)	0.0052 (4)	−0.0078 (4)	0.0030 (4)
C1	0.039 (2)	0.052 (3)	0.045 (2)	0.000	0.0039 (18)	0.000
C2	0.0429 (16)	0.0472 (16)	0.0393 (14)	0.0059 (15)	−0.0087 (14)	0.0035 (12)
C3	0.0513 (19)	0.0522 (18)	0.0431 (16)	−0.0068 (16)	−0.0077 (14)	0.0116 (14)
C4	0.046 (3)	0.074 (3)	0.040 (2)	0.000	0.0090 (19)	0.000
C5	0.066 (2)	0.0448 (16)	0.0580 (17)	0.0110 (16)	−0.0093 (16)	0.0050 (15)
C6	0.0420 (17)	0.0402 (15)	0.0435 (14)	−0.0036 (13)	−0.0045 (13)	−0.0030 (13)
C7	0.0382 (16)	0.0395 (15)	0.0473 (16)	−0.0037 (13)	0.0010 (13)	0.0034 (13)
C8	0.0489 (19)	0.0495 (17)	0.0445 (16)	−0.0017 (15)	−0.0083 (14)	0.0061 (14)
C9	0.058 (2)	0.0524 (18)	0.0372 (15)	−0.0095 (15)	−0.0059 (14)	−0.0013 (14)
C10	0.066 (2)	0.0489 (18)	0.0533 (18)	−0.0071 (16)	0.0021 (16)	−0.0089 (16)
C11	0.075 (2)	0.0375 (16)	0.068 (2)	0.0045 (16)	0.0041 (18)	−0.0011 (16)
C12	0.063 (2)	0.0460 (17)	0.0586 (19)	0.0035 (16)	−0.0019 (17)	0.0070 (15)
C13	0.0508 (18)	0.0431 (16)	0.0483 (16)	0.0008 (14)	−0.0011 (14)	0.0020 (14)
C14	0.0413 (16)	0.0360 (14)	0.0404 (15)	−0.0049 (13)	0.0017 (12)	0.0015 (12)
C15	0.0492 (18)	0.0385 (15)	0.0400 (15)	−0.0085 (14)	0.0037 (13)	−0.0004 (12)

### Geometric parameters (Å, º)

**Table:**

S1—C7	1.757 (3)	C7—C8	1.419 (4)
S1—C5	1.805 (3)	C8—C9	1.346 (4)
C1—C2	1.378 (3)	C8—H8	0.9300
C1—C2^i^	1.378 (3)	C9—C15	1.414 (4)
C1—H1	0.9300	C9—H9	0.9300
C2—C3	1.384 (4)	C10—C11	1.361 (4)
C2—C5	1.501 (4)	C10—C15	1.406 (4)
C3—C4	1.371 (3)	C10—H10	0.9300
C3—H3	0.9300	C11—C12	1.390 (4)
C4—C3^i^	1.371 (3)	C11—H11	0.9300
C4—H4	0.9300	C12—C13	1.361 (4)
C5—H5A	0.9700	C12—H12	0.9300
C5—H5B	0.9700	C13—C14	1.414 (4)
C6—C7	1.365 (4)	C13—H13	0.9300
C6—C14	1.410 (3)	C14—C15	1.416 (3)
C6—H6	0.9300		
			
C7—S1—C5	103.79 (13)	C9—C8—C7	121.4 (3)
C2—C1—C2^i^	121.4 (4)	C9—C8—H8	119.3
C2—C1—H1	119.3	C7—C8—H8	119.3
C2^i^—C1—H1	119.3	C8—C9—C15	121.1 (3)
C1—C2—C3	118.9 (3)	C8—C9—H9	119.5
C1—C2—C5	120.5 (3)	C15—C9—H9	119.5
C3—C2—C5	120.5 (3)	C11—C10—C15	121.2 (3)
C4—C3—C2	120.1 (3)	C11—C10—H10	119.4
C4—C3—H3	120.0	C15—C10—H10	119.4
C2—C3—H3	120.0	C10—C11—C12	120.1 (3)
C3^i^—C4—C3	120.7 (4)	C10—C11—H11	119.9
C3^i^—C4—H4	119.6	C12—C11—H11	119.9
C3—C4—H4	119.6	C13—C12—C11	120.5 (3)
C2—C5—S1	109.20 (19)	C13—C12—H12	119.7
C2—C5—H5A	109.8	C11—C12—H12	119.7
S1—C5—H5A	109.8	C12—C13—C14	121.0 (3)
C2—C5—H5B	109.8	C12—C13—H13	119.5
S1—C5—H5B	109.8	C14—C13—H13	119.5
H5A—C5—H5B	108.3	C6—C14—C13	122.5 (2)
C7—C6—C14	121.4 (2)	C6—C14—C15	119.3 (2)
C7—C6—H6	119.3	C13—C14—C15	118.2 (2)
C14—C6—H6	119.3	C10—C15—C9	122.9 (3)
C6—C7—C8	118.6 (2)	C10—C15—C14	118.9 (3)
C6—C7—S1	126.3 (2)	C9—C15—C14	118.2 (2)
C8—C7—S1	115.1 (2)		

Symmetry code: (i) *x*, −*y*+1/2, *z*.

### Hydrogen-bond geometry (Å, º)

Cg1 and Cg2 are the centroids of the C1–C4/C2'/C3' and C6–C9/C14/C15 rings, respectively.

**Table:**

*D*—H···*A*	*D*—H	H···*A*	*D*···*A*	*D*—H···*A*
C5—H5*B*···S1^ii^	0.97	2.86	3.806 (4)	164
C1—H1···*Cg*1^ii^	0.93	2.94	3.867 (4)	173
C13—H13···*Cg*2^iii^	0.93	2.76	3.503 (3)	138

Symmetry codes: (ii) *x*+1/2, *y*, −*z*+1/2; (iii) *x*+1/2, −*y*+1/2, −*z*+1/2.
